# New Contributions to the Elimination of Chagas Disease as a Public Health Problem: Towards the Sustainable Development Goals by 2030

**DOI:** 10.3390/tropicalmed6010023

**Published:** 2021-02-11

**Authors:** Jorg Heukelbach, Andréa Silvestre de Sousa, Alberto Novaes Ramos

**Affiliations:** 1 Postgraduate Course in Public Health, School of Medicine, Federal University of Ceará, Fortaleza, CE 60430-140, Brazil; novaes@ufc.br; 2 Evandro Chagas National Institute of Infectious Diseases, Oswaldo Cruz Foundation, Rio de Janeiro, RJ 21040-360, Brazil; andrea.silvestre@ini.fiocruz.br; 3 Department of Community Health and Postgraduate Course in Public Health, School of Medicine, Federal University of Ceará, Fortaleza, CE 60430-140, Brazil

**Keywords:** Chagas disease, American trypanosomiasis, *Trypanosoma cruzi* infection, neglected tropical disease, public health, epidemiology, prevention and control, surveillance, clinical research, One Health

Despite being described for the first time more than 110 years ago, Chagas disease persists as one of the most neglected tropical diseases. There are limited new treatment options, and diagnosis, surveillance, and control face major bottlenecks. Morbidity and mortality are still high in many settings, both in endemic and non-endemic areas.

This Special Issue of *Tropical Medicine and Infectious Diseases* contains a total of 14 peer-reviewed papers (one editorial, eight original papers, three reviews, one case report, and one opinion paper). The papers span a variety of disciplines and contribute significantly to reduce the gap in scientific knowledge on Chagas disease, towards the Sustainable Development Goals by 2030 [1].

In their editorial, Simone Kropf and Nísia Lima (the current president of the Oswaldo Cruz Foundation in Rio de Janeiro—the first woman in this position) highlight the importance of the recent introduction of the 14th April as the World Chagas Disease Day [2]. On that day in 1909, human infection with *Trypanosoma cruzi* infection was first identified by Carlos Chagas in Brazil. The authors make a strong point for the importance of celebrating this day as a social symbol of science and tropical medicine, focusing on underprivileged and neglected populations in rural hinterlands, being generated not only from European universities, but also by scientists from endemic areas. In this context, the 14th April is also a call for health as a right for all, and for breaking the vicious circle of infectious diseases and poverty. This day may help to give a voice to people at risk, recently organized in an important movement: the International Federation of Associations of People Affected by Chagas Disease (http://findechagas.org/home-en/; accessed on 7 February 2021).

Some papers of this Special Issue focus on health systems and control programs, health care rights, and the burden of Chagas disease for the societies in endemic areas. In most countries, acute disease is of compulsory notification on a national level, but not chronic Chagas disease. Thus, Rocha Siriano et al. [3] highlight the importance of mandatory notification also of chronic Chagas disease to improve the surveillance and follow-up of affected people for comprehensive care. They describe the implementation process of mandatory notification of the disease in the Brazilian state of Goiás. This state lies in the endemic region, with high morbidity and mortality burdens, and has been the pioneer for compulsory notification of chronic Chagas disease since 2013. Since February 2020, chronic disease is of mandatory notification nationwide in Brazil. Premature death related to Chagas disease not only causes suffering for individuals and their families, but also a significant national economic burden, as shown by Olivera et al. [4]. They examined the potential years of work lost, and productivity costs caused by Chagas disease in Colombia, and showed the social significance, making an additional strong point for the strengthening of control programs within the realm of early diagnosis and treatment. Sagenito et al. [5] show that despite being an important public health problem, Chagas disease research is heavily underfunded. Less than 1% of funding and financial support for research on neglected tropical diseases has been allocated to Chagas disease initiatives. This paper shows that the disease is not only being neglected by society and health care workers, but also by policy makers and funding agencies. Another study from Brazil highlights the importance of social, cultural, health system access, and human rights aspects regarding Chagas disease in a highly vulnerable group—Bolivian immigrants to Brazil [6]. While in Brazil healthcare as a human right is guaranteed by the Constitution, including for immigrants, the authors show that in practice, immigrants encounter high barriers for diagnosis and treatment against Chagas disease, such as the regular residency permit, and make a call for the implementation of multidisciplinary teams considering specific social and cultural needs, based on human rights.

Other papers focus on clinical and therapeutic aspects. In their cohort study on children and adolescents from the Brazilian Amazon region, Neves Pinto et al. [7] describe the clinical features of acute and chronic Chagas disease in this population, and the positive effect of the early management of cardiac complications. Their data indicate the effectiveness of treatment for people living in outreach areas. In another paper, Hasslocher-Moreno et al. [8] describe a progression rate of about 7% from the indeterminate to the chronic form of the disease, in a 22-year cohort study, which is lower than usually expected. In an opinion paper, Mendes et al. [9] discuss the importance of the IPEC-FIOCRUZ score—a tool for identifying patients at higher risk—for the prophylaxis against cardioembolic stroke, in patients with Chagas disease. In a retrospective analysis also from Brazil, Rossi Neto et al. [10] suggest that benznidazole may be an effective prophylactic treatment against Chagas disease reactivation in immunosuppressed patients having undergone heart transplantation. Incidence of Chagas disease reactivation in patients receiving benznidazole was 11%, as compared to 46% in patients without prophylaxis. This study reinforces the need for systematic monitoring for Chagas disease reactivation after heart transplantation, and calls for specific randomized double-blinded controlled trials. A case report presents a patient with leishmaniasis and Chagas disease co-infection, and the treatment with meglumine antimoniate which modulated the patient’s immune response against both diseases positively [11].

In an entomological survey, Tustin et al. [12] focused on the infection of triatome bugs with *T. cruzi.* They showed that the prevalence of infection in bugs from residences in Peru increased with stage, and that the prevalence in bugs is associated with the number of bites.

In addition, there are three reviews in this Special Issue. First, José Luis Ramirez describes the history of the discovery of the *T. cruzi* genome about 15 years ago, the participation of Latin American researchers, and the implication for Chagas disease research, such as the elaboration of new diagnostic tools [13]. Another review focuses on the reactivation of Chagas disease after heart transplantation [14]. Thirdly, Moll-Bernardes et al. [15] review imaging modalities to detect myocardial fibrosis, inflammation and sympathetic denervation related to Chagas disease—specific factors related to ventricular arrhythmia and sudden death.

This Special Issue provides additional insights into several aspects of Chagas disease, which is still persisting as a public health problem in many Latin American countries. The recently launched new WHO roadmap (Ending the neglect to attain the Sustainable Development Goals: A road map for neglected tropical diseases 2021–2030) [1] defines Chagas disease as one of the diseases in this group targeted for elimination as a public health problem by 2030. This means that 15 countries will have to achieve interruption of transmission through the four main transmission routes (vector, transfusion, transplantation, and congenital transmission), with 75% antiparasitic treatment coverage of the target population. Faced with these new challenges, the coming years will require strong integrated approaches including different aspects, such as evidence-based guidelines, access to healthcare, and human rights [16].

The provided information in this Special Issue will contribute to better understanding several aspects of Chagas disease. The diversity of papers and the new information provided evidence the need for further investment and interdisciplinary work. In fact, Chagas disease is a paradigmatic example for the application of the multidisciplinary One Health approach, to improve its control measures and to reach the elimination goals. The disease encompasses aspects of all four One Health determinant groups related to infection and severe morbidity [17]: firstly, Chagas disease is strongly related to factors involving people and society (including poverty, social inequality, and inadequate living conditions); secondly, animal health plays an important role (such as the presence of wild animals serving as reservoirs, and destruction of natural habitats, increasing the risk of zoonotic transmission); thirdly, governance and health systems are pivotal factors (including vulnerable national and local health systems, inadequate diagnostic capabilities, insufficient surveillance systems, limited priorities of decision makers, and little research funding); and fourthly, the environment, deforestation, and climate change are additional drivers not only for vector transmission, but also for foodborne/oral transmission. Consequently, intensified operational and implementation research efforts are needed to identify the optimal interventions and bottlenecks of control programs, for each specific setting. We are pleased to share the content with the international scientific community, and dedicate this Special Issue to the most underprivileged populations suffering from this and other neglected tropical diseases in Latin America and elsewhere around the globe.

## Data Availability

Not applicable.

## References

[1] WHO (2021). Ending the Neglect to Attain the Sustainable Development Goals: A Road Map for Neglected Tropical Diseases 2021–2030.

[2] Kropf P.S., Lima T.N. (2020). The 14th of April, past and present. Trop. Med. Infect. Dis..

[3] da Rocha Siriano L., Marchiol A., Certo P.M., Cubides J.-C., Forsyth C., de Sousa A.F. (2020). Mandatory notification of chronic chagas disease: Confronting the epidemiological silence in the State of Goiás, Brazil. Trop. Med. Infect. Dis..

[4] Olivera M.J., Palencia-Sánchez F., Riaño-Casallas M. (2021). The cost of lost productivity due to premature chagas disease-related mortality: Lessons from Colombia (2010–2017). Trop. Med. Infect. Dis..

[5] Sangenito L.S., Branquinha M.H., Santos A.L.S. (2020). Funding for chagas disease: A 10-year (2009–2018) survey. Trop. Med. Infect. Dis..

[6] Aith F.M.A., Forsyth C., Shikanai-Yasuda M.A. (2020). Chagas disease and healthcare rights in the bolivian immigrant community of São Paulo, Brazil. Trop. Med. Infect. Dis..

[7] Neves Pinto A.Y.D., Valente V.D.C., Valente S.A.D.S., Motta T.A.R., Ventura A.M.R.D.S. (2020). Clinical, cardiological and serologic follow-up of chagas disease in children and adolescents from the Amazon Region, Brazil: Longitudinal study. Trop. Med. Infect. Dis..

[8] Hasslocher-Moreno A.M., Xavier S.S., Saraiva M.R., Sangenis C.L.H., de Holanda T.M., Veloso H.H., da Costa R.A., de Mendes S.N.S.F., do Brasil A.A.P.E., da Silva S.G.M. (2020). Progression rate from the indeterminate form to the cardiac form in patients with chronic chagas disease: Twenty-two-year follow-up in a Brazilian urban cohort. Trop. Med. Infect. Dis..

[9] Mendes F.D.S.N.S., Mediano M.F.F., Silva R.S., Xavier S.S., do Brasil P.E.A.A., Saraiva R.M., Hasslocher-Moreno A.M., de Sousa A.S. (2020). Discussing the score of cardioembolic ischemic stroke in chagas disease. Trop. Med. Infect. Dis..

[10] Neto R.J.M., Finger M.A., dos Santos C.C. (2020). Benznidazole as prophylaxis for chagas disease infection reactivation in heart transplant patients: A case series in Brazil. Trop. Med. Infect. Dis..

[11] Rezende-Oliveira K., Gómez-Hernández C., Silva M.V.D., de Oliveira F.R., Machado R.J., de Teixeira A.S.L., Castellano L.R.C., Correia D., Rodrigues V. (2020). Effects of meglumine antimoniate treatment on cytokine production in a patient with mucosal leishmaniasis and chagas diseases co-infection. Trop. Med. Infect. Dis..

[12] Tustin A.W., Castillo-Neyra R., Tamayo L.D., Salazar R., Borini-Mayorí K., Levy M.Z. (2020). Elucidating the mechanism of trypanosoma cruzi acquisition by triatomine insects: Evidence from a large field survey of triatoma infestans. Trop. Med. Infect. Dis..

[13] Ramirez J.L. (2020). *Trypanosoma cruzi* genome 15 years later: What has been accomplished?. Trop. Med. Infect. Dis..

[14] Moreira M.d.C.V., Cunha-Melo R.J. (2020). Chagas disease infection reactivation after heart transplant. Trop. Med. Infect. Dis..

[15] Moll-Bernardes R.J., Rosado-de-Castro P.H., Camargo G.C., Mendes F.S.N.S., Brito A.S.X., Sousa A.S. (2020). New imaging parameters to predict sudden cardiac death in chagas disease. Trop. Med. Infect. Dis..

[16] Ramos-Junior A.N., Sousa A.S. (2017). The continuous challenge of chagas disease treatment: Bridging evidence-based guidelines, access to healthcare, and human rights. Rev. Soc. Bras. Med. Trop..

[17] Heukelbach J. (2020). One health & implementation research: Improving health for all. One Health Implement. Res..

